# Genomic Characteristics of Bladder Cancer: An AACR Project GENIE Study

**DOI:** 10.3390/ijms262311653

**Published:** 2025-12-01

**Authors:** John Paul Braun, Kenneth A. D. Palattao, Elijah Torbenson, Beau Hsia, Abubakar Tauseef

**Affiliations:** 1 School of Medicine, Creighton University, Omaha, NE 68178, USA; johnpaulbraun@creighton.edu (J.P.B.); kennypalattao@creighton.edu (K.A.D.P.J.); elijahtorbenson@creighton.edu (E.T.); 2 School of Medicine, Creighton University, Phoenix, AZ 85012, USA; beauhsia@creighton.edu; 3 Department of Internal Medicine, School of Medicine, Creighton University, Omaha, NE 68178, USA

**Keywords:** bladder cancer, urothelial carcinoma, genomic profiling, AACR Project GENIE, somatic mutations, copy number alterations, demographic disparities, targeted therapy

## Abstract

Bladder and urothelial carcinoma are marked by profound genomic diversity. Using a large, multi-institutional dataset, we performed comprehensive genomic profiling of 4631 tumor samples from 4050 individuals. A retrospective analysis of bladder and urothelial cancer was performed using the AACR Project GENIE database. Demographic associations, mutation frequencies, copy number changes, and survival correlations were analyzed with a *p*-value < 0.05. Frequent mutations were identified in *TP53*, *TERT*, *KDM6A*, *KMT2D*, *ARID1A*, and *FGFR3*. Mutation frequencies varied by sex and race, with specific alterations enriched in female and Asian patients. Distinct patterns of co-occurrence, including *TP53* with *RB1*, and mutual exclusivity, including *TP53* with *FGFR3* or *KDM6A*, revealed distinct molecular subtypes. This study highlights the extensive heterogeneity of bladder cancer, and our findings emphasize the clinical importance of molecular stratification and support the need for further mechanistic and prospective studies to inform the development of targeted therapies.

## 1. Introduction

Bladder cancer, predominantly classified as urothelial carcinoma, is a malignant tumor arising from the epithelial lining of the urinary bladder and is one of the most common genitourinary malignancies worldwide [1]. According to the World Health Organization (WHO) 2022 classification, bladder tumors are categorized based on lineage into urothelial, squamous, and glandular types, with further distinction between non–muscle-invasive and muscle-invasive forms, each presenting with unique histopathological features [2]. Clinically, patients most often present with painless gross hematuria, though symptoms may also include irritative voiding and, in advanced stages, pelvic pain or urinary obstruction [3]. Five-year relative survival rates vary greatly based on stage, reaching approximately 71–78% for localized disease and declining sharply to 8–9% for metastatic cases [4].

Globally, bladder cancer accounts for around 550,000 new diagnoses and 200,000 deaths annually, with a marked male predominance and incidence rates four-fold higher in men than women [5]. The disease is primarily seen in older adults, with the median age at diagnosis near 73 years [6]. Notable geographic variation exists, with higher rates observed in Eastern Asia, North America, and Western Europe. Established risk factors include tobacco smoking, occupational exposure to carcinogenic chemicals, chronic bladder inflammation, and certain genetic predispositions [7]. Additionally, incidence and outcomes can vary by ethnicity, with recent epidemiological studies highlighting disparities among racial and geographic groups [8].

Diagnosis of bladder cancer typically involves urine cytology, cystoscopic evaluation, and histological confirmation via transurethral resection or targeted biopsy [9]. Imaging, such as CT urography or MRI, aids in staging and assessment of tumor spread. Management strategies are stage-dependent: non–muscle-invasive cancers are often treated with transurethral resection and intravesical therapy, while muscle-invasive disease warrants radical cystectomy, chemotherapy, and may include radiotherapy or immune checkpoint inhibitors [4]. *FGFR* inhibitors and other targeted therapies have been increasingly employed in selected molecular subtypes [10]. Despite therapeutic advances, prognosis remains guarded in advanced cases, and recurrence rates are substantial.

On the molecular level, bladder cancer is characterized by significant genetic heterogeneity. Key driver alterations include mutations in *TP53*, *FGFR3*, *KDM6A*, and *CDKN2A*, along with frequent copy number changes [1]. The existence of molecular subtypes with distinct mutational profiles and pathway activations—such as those involving cell cycle regulation, chromatin modification, and receptor tyrosine kinase signaling—is now well-established. However, gaps persist in our understanding of the genetic underpinnings across different demographic groups and the broader population.

To address these needs, this study undertakes a large-scale genomic characterization of bladder and urothelial carcinoma using a diverse patient cohort. By using the AACR Project GENIE database to map mutation frequencies, demographic and molecular subtype differences, and patterns of genetic co-occurrence or exclusivity, this study aims to characterize the somatic landscape of bladder cancer. This characterization may inform the future development of molecularly tailored therapeutics, improve prognostic markers, and support future treatment guidelines.

## 2. Results

### 2.1. Demographic Summary

Patient demographics for bladder and urothelial carcinoma are shown in Table 1. This study compiled 4631 samples from 4050 patients. Of the patients represented, 3037 (75%) were male and 960 (23.7%) were female. The cohort comprised 4631 (100%) adult patients and 0 (0%) pediatric patients. With respect to ethnicity, 3158 individuals (78%) were non-Hispanic, 195 (4.8%) were Hispanic, and 697 (17.2%) were unknown or not collected. For race, the cohort included 3142 (77.6%) White, 299 (7.4%) Other, 276 (6.8%) Unknown, 149 (3.7%) Asian, 125 (3.1%) Black, 52 (1.3%) Not collected, 6 (0.1%) Native American, and 1 (0.1%) N/A individuals. Of the samples represented, 3255 (70.3%) were from primary tumors and 1111 (24%) were from metastatic tumors, 232 (5%) were not collected, 23 (0.5%) were unspecified, and 10 (0.2%) were not applicable.

### 2.2. Most Frequent Somatic Mutations

Table 2 reflects the most common somatic mutations from the represented cohort. The most common mutations found included *TP53* (n = 2402; 51.87%), *TERT* (n = 1989; 42.95%), *KDM6A* (n = 1326; 28.63%), *KMT2D* (n = 1267; 27.36%), *ARID1A* (n = 1228; 26.52%), *FGFR3* (n = 923; 19.93%), *PIK3CA* (n = 910; 19.65%), *RB1* (n = 862; 18.61%), *CREBBP* (n = 615; 13.28%), *ERBB2* (n = 605; 13.06%), and *EP300* (n = 580; 12.52%). Based on this, *TP53* mutations were the most common with notable frequencies of mutations present in *TERT*, *KDM6A*, *KMT2D*, and *ARID1A*.

### 2.3. Most Common Copy Number Alterations

Alongside the somatic mutations identified, recurrent copy number alterations (CNAs) were found in 3808 samples, a finding summarized in Table 3. Homozygous deletions (HOMDEL) events were prevalent, particularly impacting the tumor suppressor genes *CDKN2A* (n = 728; 19.12%), *CDKN2B* (n = 701; 18.59%), *MTAP* (n = 289; 16.24%), and *RB1* (n= 87; 2.28%). Amplifications, another form of CNAs, were less common. These occurred in *CCND1*, *MDM2*, *FGF19*, and *FGF4* (n = 1301; 9.46% for all).

### 2.4. Gene Mutation Differences by Sex

When stratified by sex, male and female patients demonstrated a significant enrichment for certain mutations, findings summarized in Table 4. Mutations in *AR* (n = 52; *p* = 0.00006553), *ATRX* (n = 92; *p* = 0.00008598), and *BIRC3* (n = 32; *p* = 0.0001186) were found only within females. Male patients exhibited enrichment of mutations in *CDKN1A* (n = 452; *p* = 0.000001518) and *ERBB2* (n = 653; *p* = 0.0003001).

### 2.5. Gene Mutation Differences by Race

Table 5 summarizes mutation variations by race. Several genes were enriched among Asian patients compared to white patients. *IKZF2* was nearly ten times more likely among Asians, *CCND3* four times more likely among Asians, and *HRAS* roughly two times more likely among Asians. Conversely, *STAG3* was more likely among white patients, with zero mutations recorded among Asian patients. There were no significant differences in the mutations between Black and White and Black and Asian patients.

### 2.6. Co-Occurrence and Exclusivity Rates

Significant co-occurrence and mutual exclusivity patterns were observed among frequently mutated genes, which is summarized in Table 6. Notably, *TP53* mutations were mutually exclusive with *FGFR3* (n = 185/2361; *p* < 0.001), and *KDM6A* (n = 516/2291; *p* < 0.001), indicating potential distinct molecular subtypes. In contrast, *TP53* showed significant co-occurrence with *RB1* (n = 599/1889; *p* < 0.001), suggesting a potential collaborative role in tumorigenesis.

*KDM6A* mutations significantly co-occurred with *PIK3CA* (n = 301/1446; *p* < 0.001) and *EP300* (n = 186/1338; *p* < 0.001) but were significantly exclusive with *RB1* (n = 145/1654; *p* < 0.001). *FGFR3* mutations were mutually exclusive with *ERBB2* (n = 84/1341; *p* < 0.001) but co-occurred with *PIK3CA* (n = 220/1266; *p* < 0.001), supporting a potentially targetable signaling axis. *ARID1A* showed consistent co-occurrence with multiple genes, including *ERBB2* (n = 244/1352; *p* < 0.001), *RB1* (n = 273/1436; *p* < 0.001), and *EP300* (n = 182/1252; *p* < 0.001).

### 2.7. Gene Mutations by Primary Site

Within this sample, 3255 tumors were primary while 1111 were metastatic. Notably, *TP53* and *TERT* were more common in metastatic tumors (*p* < 0.05), indicating a predilection for earlier metastasis. Conversely, *RB1*, *KDM6A*, and *KMT2D* were more common among primary tumors (*p* < 0.05). There were no significant differences in the rate of mutation among *ARID1A*, *FGFR3*, and *CDKN2A* between metastatic and primary tumors (*p* > 0.05).

## 3. Discussion

### 3.1. Study Aim and High-Level Findings

This study undertook a comprehensive genomic analysis of bladder and urothelial carcinoma, utilizing a large clinical sequencing cohort to characterize the spectrum of somatic mutations, copy number alterations, and their association with demographic factors. Our findings reveal profound genomic heterogeneity, with frequent alterations in canonical cancer pathways, and distinct mutational patterns across sex and racial subgroups. Notably, *TP53*, *TERT*, and genes encoding chromatin regulators such as *KDM6A*, *KMT2D*, and *ARID1A* emerged as some of the most commonly mutated genes in the cohort.

### 3.2. Patient Cohort and Demographic Differences

The cohort predominantly comprised male patients, mirroring the well-established increased incidence of bladder cancer among men [11]. Importantly, our study identifies several sex-specific differences in genomic alterations. For instance, mutations in *AR*, *ATRX*, and *BIRC3* were exclusively observed in female patients while males more frequently harbored mutations in *CDKN1A* and *ERBB2*. These findings are consistent with prior studies showing that bladder tumors from males have higher frequencies of mutations in *TP53*, whereas *KDM6A* is more commonly mutated in females [12]. The elevated mutation rate in *KDM6A* among women is of particular interest given its location on the X chromosome and its role in providing a “two-hit” vulnerability due to incomplete X-inactivation and may partly explain sex-based differences in tumor biology and outcomes [13]. It is established that such differences in mutational spectra likely contribute to sex disparities in clinical behavior and therapeutic responses in bladder cancer. Our data reinforce the need for further investigation into sex-specific mutational drivers as potential therapeutic targets.

Analysis of racial differences revealed a cohort composition reflecting known epidemiologic trends in bladder cancer, with a predominance of White patients and a smaller proportion of Asian and Black patients [14]. Mutational enrichment was observed for *IKZF2*, *CCND3*, and *HRAS* in Asian patients, while *STAG3* alterations were notably more common among Whites. Previous research supports the lower frequency of *TP53* and *RB1* mutations and higher prevalence of *FGFR3* and *PPARG* alterations in Asian patients compared to other groups [15]. These differences might be attributable to a combination of germline genetic architecture and diverse environmental exposures, such as carcinogen profiles for various races. However, in concordance with prior large-scale studies, no significant mutation differences were observed between Black and White patients in major DNA repair genes [15]. Our findings further support the presence of ancestry-related mutational landscapes and highlight the importance of recruiting more diverse populations to better understand potential biological and clinical disparities in bladder cancer.

### 3.3. Frequently Mutated Genes and Canonical Pathways

Our analysis underscores the vast genetic heterogeneity of bladder and urothelial carcinomas, with frequent mutations identified in widely recognized driver genes such as *TP53* (51.9%), *TERT* (42.9%), *KDM6A* (28.6%), *KMT2D* (27.4%), and *ARID1A* (26.5%), as well as notable copy number alterations including deletion of *CDKN2A/B* and amplification of *CCND1* and *MDM2*. These findings strongly align with previous genomic studies and The Cancer Genome Atlas (TCGA) reports, which also document high rates of mutations in cell cycle regulators, chromatin remodeling genes, and *TERT* promoter alterations in both muscle-invasive and non–muscle-invasive bladder cancers [16,17].

*TP53* mutations are firmly established as key drivers in high-grade and muscle-invasive bladder cancers, portending more aggressive clinical behavior and poorer prognosis [18]. Similarly, *TERT* promoter mutations are nearly ubiquitous across bladder cancer subtypes, facilitating telomerase activation and limitless replicative potential [19]. Alterations in chromatin regulatory genes such as *KDM6A*, *KMT2D*, *CREBBP*, and *EP300* are now recognized as critical contributors to tumor development, with mounting evidence that loss of histone modification and epigenetic control can enhance tumor plasticity, drive progression, and increase mutational burden [20].

In agreement with prior literature, significant copy number alterations involved deletion of *CDKN2A/B*, leading to cell cycle dysregulation, and focal amplifications in *CCND1* and *MDM2*, which are well-documented events in bladder cancer [21]. This constellation of changes highlights the centrality of cell cycle, DNA damage response, and epigenetic pathways in the molecular pathogenesis of bladder cancer.

### 3.4. Key Mutated Genes and Pathways

Mutations in *TP53* were the most frequent in our study, consistent with extensive prior research highlighting its pivotal role in high-grade tumors and association with adverse outcomes [22]. *TP53* aberrations disrupt cell cycle arrest and apoptosis, facilitating genomic instability and tumor progression. The mutual exclusivity observed between *TP53* and *FGFR3* or *KDM6A* mutations in our study is in concordance with other reports and supports the existence of molecular subtypes with distinct biology and potential therapeutic vulnerabilities [21].

Chromatin regulation genes, including *KDM6A*, *KMT2D*, *EP300*, and *ARID1A*, show high rates of mutation in bladder cancer, as demonstrated here and in multiple cohort studies [16]. Functional studies illustrate that loss of *KDM6A* or *KMT2D* promotes tumor growth and may sensitize cells to specific targeted therapies such as *EZH2* inhibitors [23]. Likewise, *ARID1A* mutations correlate with higher tumor mutational burden and may predict immunotherapy response, providing a potential guide for further development of subtype-tailored therapies [24].

Mutations in *PIK3CA*, *FGFR3*, and *ERBB2* highlight the relevance of the receptor tyrosine kinase (RTK)–RAS–PI3K signaling cascade in urothelial cancer pathogenesis, in line with previous studies documenting PI3K pathway alterations in 20–30% of bladder cancers [25]. These findings support the ongoing clinical trials employing *FGFR* inhibitors, PI3K/AKT/mTOR pathway inhibitors, and *HER2*-targeted agents.

### 3.5. Co-Occurrence and Mutual Exclusivity Patterns

Patterns of significantly co-occurring and mutually exclusive mutations emerged in our analysis, most notably mutual exclusivity between *TP53* and *FGFR3* (and between *TP53* and *KDM6A*) and co-occurrence of *TP53* with *RB1*. These relationships parallel previous observations that *TP53* and *RB1* co-mutations drive more aggressive, stem cell-like bladder cancer phenotypes [26]. Conversely, the exclusivity of *TP53* and *FGFR3* (*KDM6A*), a finding consistent with previous literature, supports a model in which differing oncogenic events define separate molecular subtypes (e.g., luminal-papillary vs. basal-squamous) [27]. Additional co-occurrence patterns were identified between *KDM6A* and *PIK3CA* and *ARID1A* and *ERBB2*, which may have provided guidance for the development of combination targeted therapies.

### 3.6. Genomic Differences: Primary Versus Metastatic Disease

Comparison of primary and metastatic bladder tumors revealed higher rates of *TP53* and *TERT* alterations in metastases, while *RB1*, *KDM6A*, and *KMT2D* mutations were more prevalent in primary tumors. This finding is in line with recent reports indicating that *TP53* mutations accumulate and are enriched during progression to advanced, metastatic disease [28].

### 3.7. Study Limitations

Several limitations must be acknowledged. First, the database approach does not permit integration of transcriptomic or epigenomic data, thus restricting analyses to DNA-level alterations and precluding evaluation of functional pathway activation [29]. Second, the lack of comprehensive clinical and treatment annotation within the dataset makes it impossible to directly correlate genomic findings with therapy response or patient survival, hindering predictive biomarker discovery. Third, the aggregation of samples from multiple centers using different sequencing technologies may introduce systematic biases, despite efforts to standardize variant calling. The absence of information regarding tumor microenvironmental markers, such as immune cell infiltration, constrains our ability to relate genomic context to immunotherapy outcomes. Moreover, because single time-point data was used, evolutionary relationships between primary and metastatic tumors within individual patients could not be assessed.

Targeted gene panels may cause bias by reporting genes as wild type when they are not included in the panel and thus are not assayed. This limitation was minimized in this study by focusing primarily on the 10 most commonly mutated genes, which were covered in nearly all panels. Furthermore, mutation frequencies were calculated using the total number of patients rather than the number of samples assessed per gene, helping to reduce overestimation for genes evaluated in only a subset of cases. Finally, the study’s cross-sectional design and modest representation of racially diverse subgroups limit the generalizability of ancestry-specific findings and underscore the need for larger, more balanced cohorts in future work.

## 4. Materials and Methods

This study was provided institutional review board exemption by Creighton University (Omaha, NE, USA) due to its use of publicly available, de-identified data from American Association for Cancer Research (AACR) Project Genomics Evidence Neoplasia Information Exchange (GENIE)^®^ database. Data was retrieved on 15 July 2025 using cBioPortal (v17.0-public) online software, which includes genomic and clinical data from 2017 to present.

The AACR GENIE^®^ database compiles genomic sequencing data from 19 cancer centers worldwide. The dataset reflects variability in the sequencing methods used, which include whole-genome sequencing (WGS), whole-exome sequencing (WES), and targeted gene panels ranging in size from 50 to 555 genes. Among all samples, around 80% were analyzed using targeted panels, 15% with WES, and 5% with WGS. Each platform demonstrated distinct sequencing depths: targeted panels typically reached over 500× coverage, WES averaged about 150×, and WGS achieved approximately 30×. Of the total samples, 65% were derived from tumor-only sequencing, while 35% included matched tumor-normal pairs, enabling the exclusion of germline variants.

Although each participating institution in the GENIE consortium uses its own pipeline for mutation calling and annotation, they follow the GENIE harmonization guidelines, as outlined by Genome NEXUS. Tools such as GATK for variant calling and ANNOVAR for annotation are commonly used, with specific versions and configurations differing by institution. Clinical outcome and therapeutic response data are available for certain types of cancer within the dataset, but treatment information is not included for bladder urothelial carcinoma. It’s also important to recognize that differences in bioinformatics workflows may occur both across and within institutions. Genomic analyses are conducted using either comprehensive methods like whole-genome or whole-exome sequencing or through targeted gene panels that can include up to 555 genes.

The study included patients diagnosed pathologically with bladder urothelial carcinoma, selected from a broader cohort of urological cases. Each sample was classified as either primary—originating from the original tumor site—or metastatic—collected from distant sites of disease spread. To assess differences in gene-specific mutation frequencies between primary and metastatic tumors, chi-squared tests were conducted using the proportion of mutated samples within each category.

The dataset included genomic information such as somatic mutations, along with histological classifications and clinical demographics including race, sex, and age. Although the design of targeted sequencing panels varied across institutions, most included major cancer-related genes like *KMT2D*, *TP53*, and *PIK3CA*. Genes lacking clinical actionability were typically excluded, and structural variants were not analyzed in this study.

Despite recent progress, the genetic basis of bladder urothelial carcinoma remains only partially understood. Uncovering additional genetic alterations that contribute to tumor progression, therapeutic resistance, and metastasis is crucial for improving diagnostic tools and treatment strategies. This study utilizes a publicly accessible dataset to further explore the somatic mutation profile of NPC, with the aim of guiding future approaches to therapy and early detection.

Copy number alterations (CNAs) were analyzed with an emphasis on identifying both amplifications and homozygous deletions, and the frequency of recurrent events was determined. Tumor mutational burden (TMB) was calculated as the number of somatic mutations—both synonymous and nonsynonymous—per megabase of DNA sequenced. For targeted panels, TMB was adjusted by dividing the total mutation count by the size of the panel (e.g., total mutations divided by 1.5 for a 1.5 Mb panel). To approximate whole-exome sequencing (WES)-equivalent TMB, these panel-based values were further refined using linear regression models developed by the AACR Project GENIE consortium. These models account for panel size and other relevant variables to correct for discrepancies across sequencing platforms and improve cross-study comparability. WES-equivalent TMB estimates are available upon request from GENIE [30].

Gene mutation frequencies were calculated by dividing the number of affected patients by the total patient count in the cohort, rather than by the number of samples tested for each individual gene. This approach prevented inflation of mutation rates for genes evaluated in a limited subset of samples. For copy number alterations and structural variants, frequencies were calculated using the total number of samples analyzed for each respective alteration.

Samples with incomplete data were excluded from all analyses. Statistical evaluations were conducted using the cBioPortal website and R and RStudio version 4.4.2 (R Foundation for Statistical Computing, Boston, MA, USA), with significance set at a *p*-value threshold of <0.05. Continuous variables are reported as mean ± standard deviation (SD), while categorical variables are summarized as counts and percentages. Associations involving categorical variables were analyzed using the chi-squared test. For continuous variables, normality was assessed prior to group comparisons; normally distributed data were evaluated with two-sided Student’s *t*-tests, whereas non-normally distributed data were compared using the Mann–Whitney U test. To account for multiple testing, the Benjamini–Hochberg method was applied to control the false discovery rate (FDR).

Somatic mutation data were filtered to include only nonsynonymous variants—such as missense, nonsense, frameshift, and splice-site mutations—with a variant allele frequency (VAF) of at least 5% and a minimum sequencing depth of 100×. Synonymous mutations and variants of uncertain significance were excluded from the analysis. Mutation information was obtained from the harmonized annotation files provided by the GENIE consortium, which offer standardized variant descriptions, including gene names and corresponding protein alterations, across all contributing institutions.

## Figures and Tables

**Table 1:** Bladder cancer patient demographics.

Demographics	Category	n (%)
Sex	Male	3037 (75)
	Female	960 (23.7)
	Unknown	53 (1.3)
Age category	Adult	4631 (100)
	Pediatric	0 (0)
Ethnicity	Non-Hispanic	3158 (78)
	Unknown/Not Collected	697 (17.2)
	Hispanic	195 (4.8)
Race	Asian	149 (3.7)
	White	3142 (77.6)
	Black	125 (3.1)
	Other	299 (7.4)
	Unknown	276 (6.8)
	Not collected	52 (1.3)
	Native American	6 (0.1)
	N/A	1 (0.1)
Sample Type	Primary	3255 (70.3)
	Metastasis	1111 (24)
	Not Collected	232 (5)
	Unspecified	23 (0.5)
	Not applicable	10 (0.2)

**Table 2:** Most frequently mutated genes.

Gene	Number of Mutations	Frequency (%)
*TP53*	3046	65.8
*TERT*	2137	46.1
*KMT2D*	1921	41.5
*ARID1A*	1575	34.0
*KDM6A*	1448	31.3
*FGFR3*	1037	22.4
*PIK3CA*	1025	22.1
*RB1*	986	21.3
*ERBB2*	757	16.3
*CREBBP*	740	16.0

**Table 3:** Most common copy number alterations.

Gene	Cytoband	CNA	Number	Frequency
*CDKN2A*	9p21.3	HOMDEL	728	19.1
*CDKN2B*	9p21.3	HOMDEL	701	18.6
*CCND1*	11q13.3	AMP	378	10.0
*MDM2*	12q15	AMP	341	9.0
*FGF19*	11q13.3	AMP	296	9.6
*MTAP*	9p21.3	HOMDEL	289	16.2
*FGF4*	11q13.3	AMP	286	9.3
*E2F3*	6p22.3	AMP	283	9.9
*FGF3*	11q13.3	AMP	269	8.7
*ERBB2*	17q12	AMP	255	6.7
*SDHC*	1q23.3	AMP	213	5.6
*RB1*	13q14.2	HOMDEL	87	2.28

**Table 4:** Gene mutation differences by sex.

Gene (Chi-Squared) Mutations	Male, n (%)	Female, n (%)	*p*-Value
*CDKN1A*	452 (13.69)	83 (8.18)	*p* = 0.000001518
*AR*	81 (2.4)	52 (4.99)	*p* = 0.00006553
*ATRX*	180 (5.33)	92 (8.82)	*p* = 0.00008598
*BIRC3*	41 (1.78)	32 (4.51)	*p* = 0.0001186
*ERBB2*	653 (18.71)	151 (13.96)	*p* = 0.0003001

**Table 5:** Gene mutation differences by race.

Gene (Chi-Squared) Mutations	Asian, n (%)	White, n (%)	*p*-Value
*STAG3*	0 (0)	2 (28.6)	*p* = 0.0013
*IKZF2*	7 (18.92)	4 (1.99)	*p* = 0.0003
*CCND3*	9 (5.23)	52 (1.48)	*p* = 0.0018
*HRAS*	14 (8.00)	114 (4.16)	*p* = 0.0021

**Table 6:** Co-occurrence and exclusivity rates.

Gene A	Gene B	Neither	A Not B	B Not A	Both	Log2 Odds Ratio	*p*-Value	q-Value	Tendency
*TP53*	*FGFR3*	1000	1563	613	185	−2.373	<0.001	<0.001	Mutual exclusivity
*TP53*	*RB1*	1472	1149	141	599	2.44	<0.001	<0.001	Co-occurrence
*FGFR3*	*RB1*	1865	756	698	42	−2.75	<0.001	<0.001	Mutual exclusivity
*KDM6A*	*FGFR3*	1878	665	424	374	1.27	<0.001	<0.001	Co-occurrence
*KDM6A*	*RB1*	1707	914	595	145	−1.14	<0.001	<0.001	Mutual exclusivity
*KDM6A*	*PIK3CA*	1915	758	387	301	0.98	<0.001	<0.001	Co-occurrence
*FGFR3*	*ERBB2*	2020	714	543	84	−1.19	<0.001	<0.001	Mutual exclusivity
*ARID1A*	*ERBB2*	2009	725	383	244	0.82	<0.001	<0.001	Co-occurrence
*FGFR3*	*PIK3CA*	2095	578	468	220	0.77	<0.001	<0.001	Co-occurrence
*ARID1A*	*RB1*	1925	696	467	273	0.69	<0.001	<0.001	Co-occurrence

## Data Availability

Data derived from public domain resources. These data were derived from the following resources available in the public domain: https://genie.cbioportal.org/, accessed on 15 June 2025.

## Abbreviations

The following abbreviations are used in this manuscript:

AACR	American Association for Cancer Research
GENIE	Genomics Evidence Neoplasia Information Exchange
cBioPortal	Cancer Bioinformatics Portal
WES	Whole-exome sequencing
WGS	Whole-genome sequencing
CNAs	Copy number alterations
SD	Standard deviation
FDR	False discovery rate
TMB	Tumor mutational burden
VAF	Variant allele frequency
TP53	Tumor Protein P53
KDM6A	Lysine Demethylase 6A
KMT2D	Lysine Methyltransferase 2D
ARID1A	AT-Rich Interaction Domain 1A
FGFR3	Fibroblast Growth Factor Receptor 3
PIK3CA	Phosphatidylinositol-4,5-Bisphosphate 3-Kinase Catalytic Subunit Alpha
CDKN2A	Cyclin Dependent Kinase Inhibitor 2A
CDKN2B	Cyclin Dependent Kinase Inhibitor 2B
RB1	Retinoblastoma 1
ERBB2	Erb-B2 Receptor Tyrosine Kinase 2
TERT	Telomerase Reverse Transcriptase
CREBBP	CREB Binding Protein
EP300	E1A Binding Protein p300
MTAP	Methylthioadenosine Phosphorylase
CCND1	Cyclin D1
MDM2	Mouse Double Minute 2 homolog
STAG3	Stromal Antigen 3
HRAS	Harvey Rat Sarcoma Viral Oncogene
ATRX	ATRX, chromatin remodeler
BIRC3	Baculoviral IAP Repeat Containing 3
AR	Androgen Receptor
IKZF2	IKAROS Family Zinc Finger 2
TCGA	The Cancer Genome Atlas Program
PI3K/AKT/mTOR	Phosphatidylinositol 3-kinase/Protein Kinase B (AKT)/Mechanistic Target of Rapamycin Signaling Pathway
(RTK)–RAS–PI3K	Receptor tyrosine kinase–RAS–phosphatidylinositol-3-kinase pathway
WHO	World Health Organization
PPARG	Peroxisome Proliferator-activated Receptor Gamma
FGFR	Fibroblast Growth Factor Receptor
CCND3	Cyclin D3
CDKN1A	Cyclin Dependent Kinase Inhibitor 1A
SDHC	Succinate Dehydrogenase Complex Subunit C
AMP	Amplification
E2F3	E2F Transcription Factor 3
FGF3	Fibroblast Growth Factor 3
GATK	Genome Analysis Toolkit
EZH2	Enhancer of Zeste Homolog 2
HOMDEL	Homozygous Deletion
FGF19	Fibroblast Growth Factor 19
FGF4	Fibroblast Growth Factor 4
